# Confidence-Guided Fusion for Self-Supervised Monocular Depth Estimation in Endoscopy

**DOI:** 10.3390/s26134033

**Published:** 2026-06-25

**Authors:** Shuang Li, Hongbo Wang, Zhaoxu Hu, Tian Chu, Yingping Li, Liang Zhao

**Affiliations:** 1 School of Information and Artificial Intelligence, Xi’an University of Architecture and Technology Huaqing College, Xi’an 710043, China; lishuang_1219@163.com; 2 School of Mechanical and Electrical Engineering, Xi’an University of Architecture and Technology Huaqing College, Xi’an 710043, China; 3 School of Artificial Intelligence, Xidian University, Xi’an 710071, China; liyingping@xidian.edu.cn; 4 College of Information and Control Engineering, Xi’an University of Architecture and Technology, Xi’an 710055, China

**Keywords:** monocular depth estimation, confidence-guided fusion, endoscopy, self-supervised learning

## Abstract

Accurate monocular depth estimation (MDE) is a foundational task in endoscopic surgery, critical for augmenting depth perception and aiding surgical navigation. While diffusion-based and discriminative depth estimators demonstrate complementary strengths, they also exhibit asymmetric errors: discriminative models yield precise geometric boundaries but struggle in homogeneous or saturated areas, whereas diffusion models recover fine textures at the cost of occasional structural incoherence. To systematically exploit this complementarity, we present CoDepth, a novel framework that leverages confidence-guided fusion to harmonize the outputs of these heterogeneous estimators. Its core components include a complementary map extractor that identifies structured disparity disagreements, a cross-attention module for context-aware feature integration, and a probabilistic confidence network that generates spatially adaptive fusion weights. Extensive evaluations on the SCARED dataset show that CoDepth achieves improved overall performance relative to strong single-model baselines, with the most consistent gains observed in Abs Rel and δ-based accuracy, while changes in some other error metrics are more modest. Furthermore, CoDepth exhibits encouraging cross-domain generalization. When a model trained on SCARED is directly evaluated on SERV-CT, Hamlyn, and C3VD without fine-tuning, it achieves competitive performance and improves several key metrics across datasets. The framework also demonstrates enhanced robustness against common synthetic corruptions like low-light conditions, Gaussian noise, and impulse noise, underscoring its practical utility in complex clinical settings. These results suggest that confidence-guided complementary fusion provides a practical integration-level paradigm for combining heterogeneous endoscopic depth estimators.

## 1. Introduction

Deep learning has been extensively applied in various image processing domains, including image classification, object detection, image segmentation, image generation, and image captioning [1]. In particular, in medical image processing, it enables automated analysis of X-ray images, CT scans, and magnetic resonance data to assist physicians in the detection of abnormalities and disease diagnosis, among other applications. Monocular depth estimation is a fundamental task in medical image processing, aimed at predicting the depth of each pixel from a single RGB image. With rapid advances in deep learning, this technology has found applications in various fields, including augmented reality [2], scene understanding [3], and minimally invasive surgery [4]. In medical image processing, particularly in endoscopic surgery, monocular depth estimation is vital to helping surgeons understand the relative distances between objects within the endoscopic field of view. This capability aids in precise location identification and navigation within the human body. Accurate depth information enables physicians to assess spatial relationships between organ tissues and surgical instruments, reducing accidental injuries and improving surgical outcomes.

However, depth estimation in endoscopic settings presents challenges that distinguish it from traditional depth estimation tasks. A primary issue is the scarcity of real-world depth data, as acquiring ground truth is often impractical due to the invasive nature of surgeries. This constraint necessitates the adoption of self-supervised or unsupervised learning techniques. Additionally, the dynamic nature of soft tissues, with frequent deformations and movements, violates the assumptions of the static scene typically used in traditional methods. The endoscopic environment is further complicated by unstable lighting, specular highlights, and limited texture, all of which diminish the reliability of photometric cues. Moreover, the narrow field of view of endoscopic cameras, combined with frequent motion blur, limits the spatial context and presents challenges for accurate depth estimation.

To overcome these challenges, researchers have proposed innovative solutions leveraging deep learning and computer vision advancements. Both discriminative and generative paradigms have proven effective in depth estimation tasks. The discriminative paradigm focuses on learning a direct mapping between inputs and outputs, where models predict depth information from input images. In endoscopy, refs. [4,5,6,7] have achieved promising results with this paradigm. The generative paradigm, on the other hand, models the data generation process, learning the joint distribution of inputs and outputs to generate plausible depth maps. In endoscopy, refs. [8,9,10] have also achieved significant results using generative models.

Upon reviewing previous research, we observed asymmetric error patterns between the discriminative and generative paradigms. The discriminative paradigm excels at making fast and accurate global predictions, particularly when ample labeled data is available. However, it struggles in complex or sparse data scenarios, especially when the scene contains intricate local details. Conversely, the generative paradigm excels at handling incomplete data or complex scenes, capturing local details more effectively and performing better in noisy or ambiguous environments, though it requires more computational resources for training and inference. These observations led us to hypothesize that combining the strengths of both paradigms could reduce asymmetric errors and enhance accuracy.

**Figure 1:**
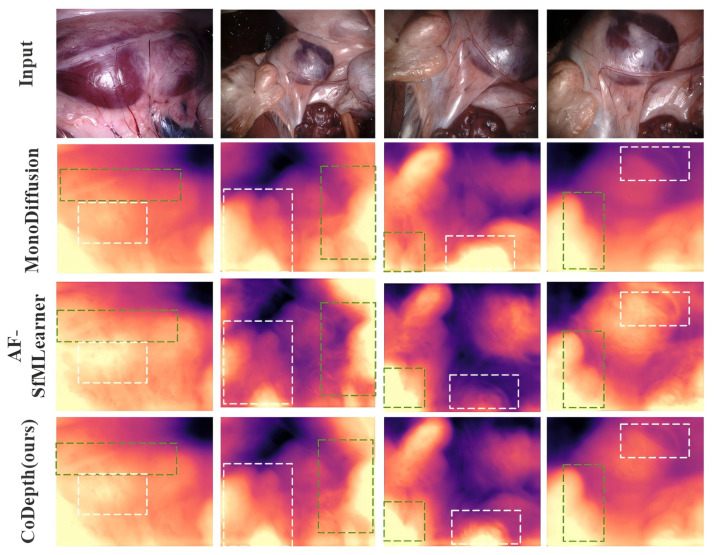
Illustration of the failure cases in MonoDiffusion [8] and AF-SfMLearner [4], highlighted with white and green boxes. In contrast, our CoDepth achieves better boundary preservation and fewer local artifacts.

To illustrate this, Figure 1 presents several failure cases of state-of-the-art monocular depth estimation methods on the SCARED dataset. As shown, the AF-SfMLearner method [4], based on the CNN-based discriminative paradigm, and the MonoDiffusion approach [8], based on the generative diffusion model paradigm, fail in different regions of the same input image. For instance, in the third column, MonoDiffusion accurately predicts the tissue boundaries within the white box, while the results from AF-SfMLearner appear blurry. On the other hand, AF-SfMLearner captures tissue boundaries within the green box, whereas MonoDiffusion fails to resolve them clearly. To mitigate these failure cases, we introduce a depth estimator that intuitively combines the strengths of both paradigms to achieve comprehensive and accurate depth estimation.

This paper introduces CoDepth, a confidence-guided complementary fusion framework for self-supervised monocular depth estimation in endoscopy. Unlike traditional single-model estimators, CoDepth explicitly models the structured disagreement between heterogeneous depth predictors and leverages this complementary information to achieve robust depth estimation under challenging surgical conditions. Our approach integrates discriminative and generative depth estimators through a suite of newly designed modules—a complementary map extractor, a 2D cross-attention fusion mechanism, a probabilistic confidence network, and a multi-scale fusion strategy. In the present implementation, the discriminative model, the diffusion model, and the auxiliary geometric modules remain frozen, while the fusion-related modules are trained in a self-supervised manner.

The core innovation of CoDepth lies in its ability to capture, interpret, and exploit model disagreement. The complementary map extractor first encodes structured disparity inconsistencies between two base predictors, revealing where each model succeeds or fails. A 2D multi-head cross-attention module then performs context-aware feature integration, aligning fine structural cues from the generative model with the globally consistent representations of the discriminative model. The fused features are further aggregated across multiple scales to enhance geometric reasoning at varying resolutions. Finally, a probabilistic confidence network predicts spatially adaptive fusion weights, guided by a multi-term confidence loss (overlap, entropy, and balance) that avoids ambiguous assignments and prevents collapse or bias, leading to stable and meaningful confidence estimation.

Through this confidence-guided complementary fusion pipeline, CoDepth effectively resolves the asymmetric error distributions that arise when deploying heterogeneous models in complex, deformable, and texture-sparse endoscopic environments. The framework adapts fusion weights at each pixel based on the estimated reliability of each predictor, enabling consistent improvements across diverse anatomical structures and imaging conditions.

The main contributions of this work are summarized as follows:

- We present CoDepth, a novel confidence-guided complementary fusion framework designed to address the asymmetric depth estimation errors commonly observed in endoscopic monocular depth estimation. CoDepth introduces a new perspective on multi-model depth estimation by explicitly modeling estimator disagreement and leveraging it through complementary feature extraction and attention-based integration.
- We design a multi-stage fusion architecture integrating Complementary Map Extraction, CrossAttention2D, Multi-scale Fusion, and Probabilistic Confidence Estimation. The Complementary Map Extractor distills structured disparity disagreements between heterogeneous depth predictors. The CrossAttention2D module performs context-aware fusion of complementary and semantic features, aligning local structural details with global depth consistency. Multi-scale fusion enhances geometric reasoning across resolutions, supporting robust predictions in complex surgical scenes. A Probabilistic Confidence Network adaptively weights each base estimator at the pixel level, trained with a multi-term confidence loss (overlap, entropy, and balance) that avoids ambiguous assignments, prevents confidence collapse or bias, and yields stable, semantically meaningful confidence maps.
- We conduct comprehensive experiments on four challenging datasets, SCARED, SERV-CT, Hamlyn, and C3VD, to validate the effectiveness of CoDepth. The results show that CoDepth achieves competitive overall performance across these benchmarks and improves several key depth metrics, with the clearest gains observed in Abs Rel and δ<1.25.

## 2. Related Work

### 2.1. Self-Supervised Depth Estimation

Monocular depth estimation (MDE) aims to infer depth from a single image. Existing methods are generally categorized into supervised and self-supervised depth estimation. Supervised methods require ground-truth depth data for model training, which can be costly due to the difficulty in obtaining real depth maps. In contrast, Self-supervised methods do not require labeled data. Instead, they generate labels during training based on the structure and characteristics of the data itself, using geometric relationships, and training the model similarly to supervised learning.

Garg et al. [11] first proposed a basic framework for training models via unsupervised learning. This framework included a convolutional neural network (CNN) for depth estimation and an image reconstruction module. Godard et al. [12] further refined this method by introducing a differentiable interpolation function, optimizing the loss function, and proposing surface-matching loss and disparity-smoothness loss, which have since become widely used in unsupervised depth estimation.

While image-pair-based methods have shown promising results, they also have notable drawbacks. For instance, they require the baseline length and camera focal length to be determined, along with images from both left and right viewpoints. These constraints limit the range of datasets available for training. To address this, researchers have proposed unsupervised methods that utilize video sequences. These methods leverage the geometric relationships between consecutive frames for training. Zhou et al. [13] pioneered a dual-network architecture, consisting of depth and pose estimation networks, which synthesize novel views via warping transformations and implement occlusion-aware masking mechanisms.

To mitigate the impact of pixel points that violate the static assumption, methods based on explainable masks [14,15] have been proposed. Johnston and Carnerio [16] enhanced self-supervised monocular depth estimation by incorporating self-attention and discrete disparity prediction, which enables better context exploration and more robust depth estimation. Liu et al. [17] introduced a self-supervised method using distillation and disparity-offset refinement to improve depth performance. Liu and Zuo [18] added a dual-attention mechanism with position and channel attention modules to capture long-range contextual information.

Further advancements include Zhang et al.’s [19] Lite-Mono, a hybrid CNN-Transformer architecture, and Yang et al.’s [20] integration of Transformer layers into CNNs to enhance texture sensitivity and use multi-head attention for pose estimation. Xu et al. [21] developed a self-supervised framework for depth and pose estimation that incorporates a Generative Latent Bank and Variational Autoencoders (VAEs). Zhu and Cui [22] introduced cosine-dissimilarity-based perceptual loss to strengthen the supervisory signals.

### 2.2. Diffusion Models

Diffusion models, as one of the leading generative models, have significantly advanced artificial intelligence by enabling the generation of high-quality data across various domains, including image synthesis, video generation, 3D creation, medical analysis, text generation, speech synthesis, time-series generation, molecular design, and graph generation. Diffusion models consist of two key processes: a forward process and a reverse process. In the forward process, noise is gradually added to the data, transforming it into a simpler distribution, typically Gaussian. The reverse process uses a trained neural network to iteratively remove this noise, recovering the original data. The network is trained using denoising score matching to predict and eliminate noise, enabling diffusion models to generate high-quality data by reversing the noise transformation.

The diffusion model has made notable advancements in the application of Monocular Depth Estimation (MDE). Saxena et al. [23] introduce DepthGen, a diffusion model for monocular depth estimation, which comprises self-supervised pre-training and supervised fine-tuning. Duan et al. [24] emphasize a denoising strategy to iteratively refine depth maps, using diffusion models to mitigate noisy depth predictions and enhance accuracy in complex environments. Their work primarily focuses on a fully supervised setting. In self-supervised MDE, Shao et al. [8] propose the MonoDiffusion framework, treating depth estimation as an iterative denoising process. This method eliminates the need for real depth labels by using pseudo-labels and teacher models for self-supervised training. Furthermore, the integration of transformers with diffusion models has shown significant promise in scaling depth estimation models. Peebles and Xie [25] introduced diffusion transformers, a scalable architecture that enhances depth-estimation accuracy by leveraging transformers to generate high-resolution depth maps.

## 3. Materials and Methods

### 3.1. Method Overview and Problem Formulation

Monocular depth estimation in endoscopy remains challenging due to weak texture, specular highlights, non-rigid tissue deformation, and frequent local ambiguities. In practice, different depth estimation paradigms often exhibit complementary behaviors: discriminative estimators usually provide more stable global geometric structure, whereas diffusion-based estimators may recover finer local details but can also introduce structural inconsistency in difficult regions.

Rather than proposing a new backbone depth estimator, this work addresses a different problem: how to reliably integrate two frozen heterogeneous depth estimators with complementary error patterns. Given an input endoscopic image *I*, the inherited discriminative and diffusion-based estimators produce two depth predictions,

(1) $$D^{\mathrm{disc}}=M_{\mathrm{disc}}\left(I\right),\;D^{\mathrm{diff}}=M_{\mathrm{diff}}\left(I\right).$$

**Figure 2:**
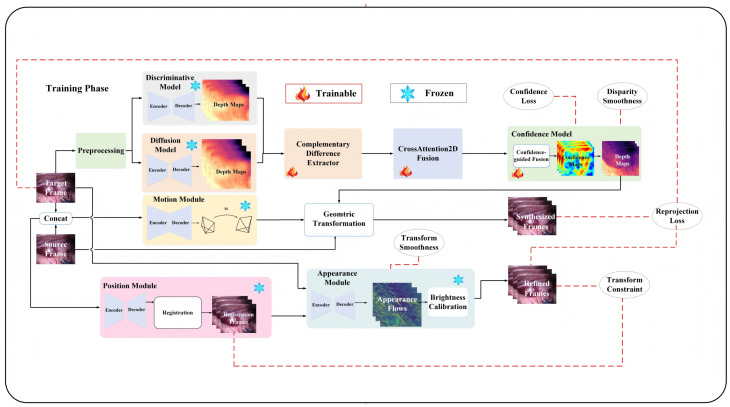
Overview of the Confidence-Guided Multi-Model Fusion framework for self-supervised monocular depth estimation. During training, the network consists of frozen components—the discriminative model, diffusion model, pose module, position module, and appearance modules—and trainable components, including the complementary feature extractor, CrossAttention2D module, and confidence prediction module. The red dashed line indicates the data loss used for self-supervised optimization.

**Figure 3:**
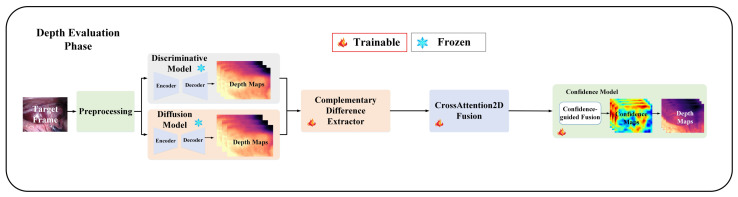
During the evaluation phase, we use the trained discriminative model, diffusion model, and confidence module to estimate the dense depth maps.

Our goal is to learn a fusion function

(2) $$D^{\mathrm{fuse}}=F\left(I,D^{\mathrm{disc}},D^{\mathrm{diff}}\right),$$

that preserves the global structural stability of the discriminative branch while selectively incorporating locally informative details from the diffusion branch. To this end, we propose a complementarity-aware confidence-guided fusion method that explicitly models inter-estimator disagreement and converts it into spatially adaptive fusion confidence. An overview of the framework is shown in Figure 2 and Figure 3.

As illustrated in Figure 2, given a target frame $I_{t}\in R^{H\times W\times 3}$, the two models infer depth in parallel. Their pixel-wise prediction discrepancy is exploited to characterize model complementarity and local uncertainty.

To estimate the reliability of the two predictions, intermediate features from both models are aggregated by a cross-attention fusion module, enabling effective interaction between discriminative and diffusion-based representations. In parallel, discrepancy cues extracted from prediction differences highlight regions with inconsistent confidence or distinct structural preferences. These cues are combined with the fused features and passed to a confidence estimation module.

The confidence estimation module predicts a dense confidence map $C\in {\left[0,1\right]}^{H\times W}$, which encodes the pixel-wise reliability of the two models. Based on *C*, a confidence-aware fusion strategy adaptively combines the two depth predictions to produce the final estimate ${\hat{D}}_{t}$, thereby improving local detail preservation and global structural consistency.

For self-supervised training, an adjacent frame is used as the source frame to provide multi-view geometric constraints. The relative pose between the source and target frames is estimated by the motion module. Given the camera intrinsic matrix *K*, the source frame is warped into the target view via a spatial transformer to generate a synthesized frame, whose appearance discrepancy from the target frame serves as a photometric supervision signal. Meanwhile, the position module estimates a registration frame from the source and target frames, and the appearance module further refines it through brightness calibration to obtain a photometrically corrected frame. These reconstructed frames, together with photometric reconstruction and geometric consistency losses, are used to optimize the depth estimation networks. The fusion-related modules are further supervised by geometric consistency and confidence regularization terms.

To avoid ambiguity between the internal branch names used in CoDepth and the external baselines reported in the experiments Table 1 summarizes the role of each branch, its relation to the corresponding baseline method, whether its weights are updated during training, and which outputs are used by the proposed fusion module.

### 3.2. Frozen Base Estimators and Inherited Supervision

To clearly separate inherited components from methodological novelty, we emphasize that the discriminative estimator, the diffusion estimator, and the auxiliary geometric supervision modules are all adopted from prior work and remain frozen in this study.

#### 3.2.1. Frozen Discriminative Model

This model adopts an encoder–decoder architecture with skip connections, where the encoder is based on ResNet-18 [26] with the fully connected layer removed. The decoder design follows that of Monodepth2 [14]. This model provides complementary structural information. All weights in this model are frozen during training, serving as a stable supervisory signal.

#### 3.2.2. Frozen Diffusion Model

The model primarily consists of two modules: a teacher module and a student module. The teacher module guides the denoising process of the student module through visual guidance conditions and knowledge distillation, leveraging its depth maps and multi-view filters. The teacher module adopts the AF-sfMLeaner [4] architecture, which achieves full-resolution depth prediction via a multi-scale approach and enhances training stability through gradient locality in the Spatial Transformer network. The student module utilizes a Lite-Mono encoder [19] to extract multi-scale features from the input images. These features are then processed through multiple 3 × 3 convolutional layers and upsampling layers to generate visual guidance conditions that steer the diffusion-based denoising process. The decoder of the student module follows the design of HRDepthDecoder [19].

#### 3.2.3. Inherited Geometric Supervision Modules

We also retain the auxiliary motion, position, and appearance modules inherited from the original self-supervised reconstruction pipeline [4]. These modules are used only to provide training-time geometric or photometric supervision.

##### Motion Module

The motion module estimates the relative camera pose between consecutive frames for geometry-based view synthesis. Let $T_{12}=\left[R,t\right]$ denote the transformation from the reference frame $I_{1}$ to the target frame $I_{2}$, where *R* and *t* are the rotation matrix and translation vector, respectively. Following [4], we adopt an encoder–decoder network to predict the 6-DoF pose from the input frame pair.

Based on the estimated pose, the reference view is warped to synthesize the target view:

(3) $$p_{2}=Rp_{1}+t.$$

Here, $p_{1}$ and $p_{2}$ denote corresponding pixel coordinates in $I_{1}$ and $I_{2}$. To handle non-grid-aligned coordinates after transformation, bilinear interpolation is applied:

(4) $$I_{2}\left(p_{2}\right)=\sum_{k}K\left(p_{2}-p_{1},k\right)\;I_{1}\left(p_{1},k\right),$$

where K(·) is the interpolation kernel and *k* indexes neighboring pixels.

##### Position Module

The position module performs dense registration between the source image $I_{s}$ and the target image $I_{t}$. It consists of an encoder–decoder architecture, where the encoder extracts multiscale geometric features from the input pair and the decoder predicts an optical flow field F together with an occlusion mask $M_{\mathrm{occ}}$.

The flow field $F\in R^{H\times W\times 2}$ encodes the pixel-wise displacement from $I_{s}$ to $I_{t}$, while the occlusion mask $M_{\mathrm{occ}}\in {\left\{0,1\right\}}^{H\times W}$ is estimated via bidirectional flow consistency:

(5) $$M_{\mathrm{occ}}=I\left({∥F_{\mathrm{forward}}-F_{\mathrm{backward}}^{-1}∥}_{1}>\tau \right),$$

where $F_{\mathrm{forward}}$ and $F_{\mathrm{backward}}$ denote the forward and backward flows, respectively, and τ is a threshold.

The source image is warped to the target view as

(6) $$I_{s\to t}=W\left(I_{s},F\right),$$

where W denotes differentiable warping with bilinear sampling. The final registered image is obtained by occlusion-aware fusion:

(7) $$I_{\mathrm{reg}}=M_{\mathrm{occ}}⊙I_{s\to t}+\left(1-M_{\mathrm{occ}}\right)⊙I_{t},$$

where ⊙ denotes element-wise multiplication.

##### Appearance Module

The appearance module models residual photometric misalignment via an appearance flow $A_{f}$. Following [4], $A_{f}$ is defined as

(8) $$A_{f}\left(p\right)=p_{2}-p_{1},$$

where $p_{1}$ and $p_{2}$ denote the corresponding pixel coordinates in $I_{1}$ and $I_{2}$, respectively.

The final refined image is obtained by flow-guided resampling of the registered image:

(9) $$I_{\mathrm{refined}}\left(p\right)=I_{\mathrm{reg}}\left(p+A_{f}\left(p\right)\right),$$

where $A_{f}$ compensates for residual appearance discrepancies between the two frames.

### 3.3. Proposed Confidence-Guided Fusion Method

The proposed method is based on the assumption that disagreement between heterogeneous estimators is not purely noise, but a structured cue reflecting local reliability differences. Accordingly, we design a trainable fusion method consisting of Complementary Map Extraction, Cross-Attention Context Fusion, and Probabilistic Confidence Prediction.

Given an input target frame $I_{t}$, the two models produce disparity predictions $d^{\mathrm{disc}}=M_{1}\left(I_{t}\right)$ and $d^{\mathrm{diff}}=M_{2}\left(I_{t}\right)$ at multiple scales. Their pixel-wise discrepancies are first exploited to obtain a complementary map. Concretely, the absolute difference between the two predictions is normalized to the range [0, 1] to derive preliminary complementary weights that highlight regions where one model is more reliable than the other. These complementary weights are then passed through a lightweight convolutional complementary extractor and a cross-attention module, which interacts with high-level fused features from both models to learn richer complementary representations. On top of these representations, a confidence prediction module outputs a two-channel confidence map

$$C=\left[C_{1},C_{2}\right]\in {\left[0,1\right]}^{2\times H\times W},$$

where $C_{1}$ and $C_{2}$ denote the per-pixel confidence for the Discriminative Model and the Diffusion Model, respectively, and satisfy $C_{1}+C_{2}=1$. The final fused depth ${\hat{d}}_{t}$ is then computed as a confidence-weighted combination of the two predictions:

(10) $${\hat{D}}_{t}=C_{1}⊙D^{\mathrm{disc}}+C_{2}⊙D^{\mathrm{diff}},$$

where $D^{\mathrm{disc}}$ represents the depth converted from the disparity $d^{\mathrm{disc}}$ estimated by the discriminative model, and $D^{\mathrm{diff}}$ represents the depth converted from the disparity $d^{\mathrm{diff}}$ estimated by the diffusion model. ⊙ denotes element-wise multiplication. The confidence maps provide a transparent view of the fusion behavior: regions where $C_{1}$ dominates are mainly influenced by the discriminative prediction, while regions where $C_{2}$ is higher rely more on the diffusion-based prediction. Moreover, a dedicated confidence regularization loss encourages non-trivial, well-balanced confidence distributions by penalizing excessive overlap between the two channels, promoting sufficient entropy, and enforcing a global balance between $C_{1}$ and $C_{2}$. This design prevents degenerate solutions where one model overwhelms the other and stabilizes the multi-model fusion during self-supervised training.

#### 3.3.1. Complementary Map Extraction

To explicitly characterize the asymmetric errors between the two predictors, we compute a normalized complementary map:

(11) $$\Delta =\frac{|d^{\mathrm{disc}}-d^{\mathrm{diff}}|}{\max(|d^{\mathrm{disc}}-d^{\mathrm{diff}}|)+\epsilon },$$

which highlights regions where the two models diverge. A lightweight convolutional extractor $\phi_{\mathrm{cmp}}$ transforms the complementary signal into a structured feature representation:

(12) $$F_{c}=\phi_{\mathrm{cmp}}\left(\left[\Delta ,\;1-\Delta \right]\right).$$

#### 3.3.2. Cross-Attention Context Fusion

To align the complementary features with semantically rich depth representations, we fuse $F_{c}$ with the multi-scale backbone features $\left\{F_{1}^{\left(k\right)},F_{2}^{\left(k\right)}\right\}$ through a 2D multi-head cross-attention module [27]:

(13) $$F_{a}=\mathrm{CrossAttn}\left(Q=W_{q}F_{c},\;K=W_{k}F_{f},\;V=W_{v}F_{f}\right),$$

where $F_{f}=\left[F_{1}^{\left(k\right)},F_{2}^{\left(k\right)}\right]$ denotes the concatenated features from both models. This operation enables the model to interpret disparity disagreement in a broader geometric and textural context, improving the reliability of confidence estimation.

To keep the additional attention cost manageable, CrossAttention2D operates on 2× downsampled feature maps and its output is then upsampled back to the original resolution. This reduces the effective sequence length by approximately 75% while preserving the structural cues required for reliable confidence estimation.

#### 3.3.3. Probabilistic Confidence Prediction

The confidence network takes the context-enhanced complementary features $F_{a}$ as input and predicts pixel-wise confidence logits $z=[z_{1},z_{2}]$, which are normalized through a softmax function:

(14) $$p_{i}=\frac{\exp(z_{i})}{\exp\left(z_{1}\right)+\exp\left(z_{2}\right)},\;i\in 1,2.$$

The resulting probability maps $p_{1}$, $p_{2}$ form the final confidence maps used for adaptive fusion. The predicted probabilities represent the model’s belief about the reliability of each depth estimator at every pixel. This formulation not only stabilizes the fusion process but also enhances interpretability: regions where diffusion-based estimators excel (e.g., low-texture surfaces) present higher $p_{2}$, while edge-rich or high-frequency regions favor $p_{1}$. The confidence maps thus encode a spatially adaptive model selection strategy learned through self-supervised optimization of the fusion branch, while the base estimators remain frozen.

#### 3.3.4. Fusion Objective and Confidence Regularization

The proposed fusion branch is designed not to simply average the predictions of two heterogeneous depth estimators, but to learn a spatially adaptive routing mechanism that selects the more reliable branch at each pixel while preserving their complementarity. In endoscopic scenes, discriminative models and diffusion-based models often fail in different regions. Therefore, an effective fusion strategy should satisfy three properties: (i) local exclusivity, to avoid unreliable averaging of conflicting predictions; (ii) decision sharpness, to produce decisive rather than ambiguous confidence assignments; and (iii) global balance, to prevent persistent dominance of one branch.

Formally, the fused depth is defined as defined in Equation (10). In the self-supervised setting, no explicit ground-truth labels are available for the confidence maps. We therefore regularize them using three complementary terms: overlap, entropy, and balance.

##### Overlap Regularization

To discourage ambiguous fusion, we penalize simultaneous high confidence from both branches at the same pixel:

(15) $$L_{ov}=\frac{1}{HW}\sum_{p}C_{1}\left(p\right)C_{2}\left(p\right).$$

This term promotes locally exclusive selection between the two predictors.

##### Entropy Regularization

To encourage decisive routing, we minimize the entropy of the confidence distribution:

(16) $$L_{ent}=-\frac{1}{HW}\sum_{p}\sum_{i=1}^{2}C_{i}\left(p\right)\log C_{i}\left(p\right).$$

Minimizing this term yields sharper confidence assignments. In practice, it is activated with a warm-up schedule for training stability.

##### Balance Regularization

To avoid global collapse to a single branch, we constrain the spatial mean confidence to remain close to a uniform distribution:

(17) $${\bar{C}}_{i}=\frac{1}{HW}\sum_{p}C_{i}\left(p\right),\;L_{bal}=\sum_{i=1}^{2}{\left({\bar{C}}_{i}-\frac{1}{2}\right)}^{2}.$$

This term acts as a weak global prior, encouraging both branches to be utilized without enforcing equal confidence at every pixel.

Together, these regularization terms enforce locally exclusive, sharp, and globally balanced confidence prediction, thereby stabilizing adaptive fusion under self-supervised training.

### 3.4. Training Objective and Optimization Strategy

Only the parameters of the proposed fusion method are optimized during training. Let

$$\Theta_{\mathrm{base}}=\left\{\Theta_{\mathrm{disc}},\Theta_{\mathrm{diff}},\Theta_{\mathrm{geo}}\right\}$$

denote the parameters of the inherited discriminative estimator, diffusion estimator, and geometric supervision modules, and let

$$\Theta_{\mathrm{fuse}}=\left\{\Theta_{\mathrm{CME}},\Theta_{\mathrm{CCF}},\Theta_{\mathrm{PCP}}\right\}$$

denote the parameters of the proposed Complementary Map Extraction, Cross-Attention Context Fusion, and Probabilistic Confidence Prediction. During training,

$$\frac{\partial L}{\partial \Theta_{\mathrm{base}}}=0,\;\frac{\partial L}{\partial \Theta_{\mathrm{fuse}}}\neq 0.$$

The overall loss function is composed of multiple terms that enforce photometric consistency, geometric constraints, and smoothness regularization across different scales. The total loss is a weighted sum of individual loss terms, averaged across multiple scales. The complete loss function is as follows:

(18) $$L_{total}=\lambda_{1}\cdot L_{D-S}+\lambda_{2}\cdot L_{R-L}+\lambda_{3}\cdot L_{T-C}+\lambda_{4}\cdot L_{T-S}+\lambda_{5}\cdot L_{conf}$$

where $L_{D-S}$ is the Disparity Smoothness Loss, $L_{R-L}$ is the Reprojection Loss, $L_{T-C}$ is the Transform Constraint Loss, $L_{T-S}$ is the Transform Smoothness Loss, $L_{conf}$ is the Confidence Loss.

- Disparity Smoothness Loss: The disparity smoothness loss encourages smooth disparity maps by penalizing large disparity changes between neighboring pixels. This helps to maintain continuity in the depth map and reduce noise. The loss function aims to minimize disparity jumps, promoting a smoother depth estimation.
  (19) $$L_{D-S}^{\left(s\right)}=\lambda_{disp}\cdot \frac{1}{2^{s}}\sum_{p}\left|\nabla d^{\left(s\right)}\right|\cdot e^{-\left|\nabla_{ref}^{\left(s\right)}\right|}$$
  where $d^{\left(s\right)}$ is the predicted disparity map, and $\lambda_{disp}$ controls the strength of the smoothness term.
- Reprojection Loss: The reprojection loss is crucial for tasks like depth estimation, stereo matching, or 3D reconstruction. It ensures that transformed or refined pixels in the image correspond closely to their target positions in the reference image, ensuring pixel-level accuracy.
  (20) $$L_{R-L}=\sum_{s\in S}\lambda_{s}\left(\alpha \cdot SSIM\left(I_{t},I_{t}^{s}\right)+\left(1-\alpha \right)\cdot {∥I_{t}-I_{t}^{s}∥}_{1}\right)$$
  Here, SSIM (Structural Similarity Index) measures the similarity between the predicted and target images, with α balancing the two terms.
- Transform Constraint Loss: The transform constraint loss ensures that the refined image closely matches the registered image after transformation. This regularizes the transformation process to ensure consistency across the predicted outputs.
  (21) $$L_{T-C}=\sum_{s\in S}\gamma_{s}\cdot {∥I_{refined}^{s}-I_{reg}^{s}∥}_{1}$$
  $\gamma_{s}$ is a scaling factor that controls the weight of this loss term.
- Transform Smoothness Loss: The transform smoothness loss penalizes abrupt changes in brightness after transformation. It ensures that the brightness variation is smooth, avoiding unnatural or sharp transitions in the transformed images.
  (22) $$L_{T-S}^{\left(s\right)}=\frac{\lambda_{smooth}}{2}\sum_{f\in F}L_{bright}\left(T^{\left(s,f\right)},I_{ref}^{\left(s\right)},I_{reg}^{\left(s,f\right)},M_{occ}^{\left(f\right)}\right)$$
  where $\lambda_{smooth}$ is a weight that controls the strength of the smoothness regularization, and $L_{bright}$ penalizes sudden changes in brightness during the transformation.
- Confidence Loss: Based on the above regularization design, the overall confidence loss is defined as
  (23) $$L_{conf}=\lambda_{ov}L_{ov}+\lambda_{ent}L_{ent}+\lambda_{bal}L_{bal},$$
  where $\lambda_{ov}$, $\lambda_{ent}$, and $\lambda_{bal}$ are weighting coefficients for the overlap, entropy, and balance terms, respectively.

### 3.5. Summary of Methodological Novelty

In summary, the novelty of this work lies in the proposed *complementarity-aware confidence-guided fusion method*, including: (i) explicit modeling of structured disagreement between two frozen heterogeneous depth estimators, (ii) context-aware interaction between complementary cues and estimator features, and (iii) probabilistic confidence-based adaptive depth integration. In contrast, the backbone estimators and auxiliary geometric supervision modules are inherited from prior work and used as frozen components.

## 4. Results and Discussion

### 4.1. Experimental Setup

#### 4.1.1. Datasets

We conduct extensive experiments on four publicly available surgical datasets.

- **SCARED** [28]. The SCARED dataset comprises 35 endoscopic videos captured using a Da Vinci Xi endoscope, showcasing fresh porcine cadaver abdominal anatomy. It includes point cloud data and ego-motion ground truth.
- **SERV-CT** [29]. The SERV-CT dataset contains 16 pairs of stereo images from two sets of porcine samples. Each sample group provides complete camera intrinsic and extrinsic calibration parameters, depth maps, disparity maps, and occlusion region annotations. The dataset addresses the challenge of validating 3D reconstruction algorithms in surgical endoscopic scenes, especially in scenarios with issues such as the absence of distinct corner features, highly reflective surfaces, and the presence of blood and smoke.
- **Hamlyn** (https://davidrecasens.github.io/EndoDepthAndMotion/ (accessed on 8 October 2025)).
  The Hamlyn Centre laparoscopic/endoscopic video dataset contains a rich collection of laparoscopic and endoscopic video data. These datasets record various complex surgical scenarios, including pig diaphragm anatomy, lobectomy, TECAB surgery, and more, covering visual challenges such as tissue deformation, motion caused by respiration and heartbeat, smoke blur, and interactions between tools and tissue. The dataset includes 38 videos and sub-datasets, providing researchers with high-quality real surgical video resources.
- **C3VD** (https://durrlab.github.io/C3VD/ (accessed on 8 October 2025)).
  The Colonoscopy 3D Video Dataset (C3VD) is collected using high-definition clinical colonoscopy and high-fidelity colon models, aimed at providing benchmark resources for computer vision methods in colonoscopy scenarios. The dataset includes 22 short videos and 10,015 frames, with each frame accompanied by paired ground truth depth, surface normals, optical flow, occlusion information, six-degree-of-freedom pose, coverage maps, and 3D models.

For depth estimation, we perform extensive experiments on the SCARED dataset and then apply models trained on SCARED to the SERV-CT, Hamlyn, and C3VD datasets to validate the generalization ability. Following a split protocol inspired by the Eigen–Zhou evaluation protocol from the KITTI benchmark [30], the SCARED dataset is divided into 15,351 frames for training, 1705 frames for validation, and 551 frames for testing.

#### 4.1.2. Implementation Details

CoDepth is implemented using the PyTorch library (version 2.0.1.) and trained on an NVIDIA RTX A5000 (manufactured by NVIDIA Corporation, Santa Clara, CA, USA). The system runs on Ubuntu 18.04 with CUDA 11.8.

Our optimization setup employs the Adam optimizer with a base learning rate of $1\times 10^{-3}$, default momentum parameters $\left(\beta_{1}=0.9,\beta_{2}=0.999\right)$, and no weight decay. We use a StepLR scheduler that decays the learning rate by a factor of 0.1 every 30 epochs. In our experiments, training is conducted for 25 epochs without early stopping, and model checkpoints are saved at regular intervals.

All backbone networks are initialized with pretrained weights. The discriminative model, the diffusion model, as well as the pose, position, and transform networks are kept frozen during training and operate in evaluation mode. Only the fusion-related modules are updated: (i) the complementary feature extractor that processes the normalized disparity disagreement between the two depth predictions, (ii) the cross-attention module that injects complementary cues into the fused high-level features, and (iii) the confidence prediction module that outputs the per-pixel confidence maps used for fusion. This design yields an efficient training process that leverages the strengths of multiple pretrained depth and geometric models while learning a lightweight, confidence-guided fusion mechanism.

Input images are resized so that both height and width are multiples of 32 to ensure compatibility with the encoder–decoder architectures. During training and validation, we log depth maps, confidence maps, and scalar statistics using TensorBoard, together with periodic validation on the target dataset. Through confidence-guided multi-model fusion and stable geometric supervision, CoDepth achieves accurate and robust monocular depth estimation suitable for complex real-world scenarios.

#### 4.1.3. Evaluation Metrics

The evaluation uses several metrics consistent with previous depth estimation works, as follows:

- Abs Rel: $\frac{1}{\left|D\right|}\sum_{d\in D}\frac{|d_{t}-d|}{d_{t}}$
- Sq Rel: $\frac{1}{\left|D\right|}\sum_{d\in D}\frac{{∥d_{t}-d∥}^{2}}{d_{t}}$
- RMSE: $\sqrt{\frac{1}{\left|D\right|}\sum_{d\in D}{∥d_{t}-d∥}^{2}}$
- RMSE log: $\sqrt{\frac{1}{\left|D\right|}\sum_{d\in D}{∥\log d_{t}-\log d∥}^{2}}$
- $\delta <\mathrm{thr}:\%\mathrm{of}d\mathrm{satisfies}\left(\max\left(\frac{d_{t}}{d},\frac{d}{d_{t}}\right)=\delta <\mathrm{thr}\right)for\;thr=1.25,\;1.25^{2},\;1.25^{3}$

Among these metrics, Abs Rel, Sq Rel, RMSE, and RMSE log are depth error metrics where lower values indicate better performance. The δ<1.25, $\delta <1.25^{2}$, and $\delta <1.25^{3}$ are depth accuracy metrics where higher values indicate better performance.

#### 4.1.4. Performance Metrics

To further analyze the model’s throughput performance, hardware resource requirements, computational efficiency, and real-time latency characteristics, we tested the following performance metrics:

- **FPS (Average Frames Per Second):** Average number of images processed per second during inference.
- **Inference Time (Average Batch Processing Time):** Mean time taken to process one batch (in seconds).
- **FLOPs (Floating Point Operations):** The total number of floating-point arithmetic operations required to perform a computation.
- **Trainable Parameters:** The number of trainable parameters in the fusion/adaptation modules used at inference time.
- **Total Params:** The total number of parameters involved in the forward pass at inference time, including frozen base models and trainable modules.

#### 4.1.5. Comparison Methods

The comparative methods include several typical self-supervised approaches and the diffusion model. SfMLearner [13] utilizes a self-supervised loss function, photometric consistency constraints, and joint learning of depth and motion information to improve depth estimation accuracy. Fang et al. [31] summarized best practices for CNN-based monocular depth estimation methods. DeFeat-Net [32] combines unsupervised representation learning and multi-task learning, simultaneously optimizing depth estimation and feature learning tasks. SC-SfMLearner [15] addresses common issues in traditional monocular depth estimation, such as scale ambiguity and lighting fluctuations, by introducing scale consistency and photometric consistency constraints. Monodepth2 [14] improves the image reconstruction loss function and optimizes photometric consistency. Endo-SfM [33] proposes a depth estimation and motion inference framework specifically designed for medical applications. AF-SfMLearner [4] introduced the appearance flow to account for variations in brightness patterns, establishing generalized dynamic image constraints that integrate geometric and radiometric transformations. MonoDiffusion [8] integrates the diffusion models into self-supervised monocular depth estimation. Yang et al. [20] proposes a lightweight self-supervised depth estimation network by tightly combining Convolutional Neural Networks (CNN) and Transformer, leveraging their ability to extract features at different scales.

### 4.2. Quantitative Comparison Results

CoDepth demonstrates strong and consistent performance on the SCARED, SERV-CT, Hamlyn, and C3VD datasets, surpassing or closely matching existing depth estimation methods across key metrics. As shown in Table 2, CoDepth achieves the lowest absolute relative error (Abs Rel ↓ 0.055), squared relative error (Sq Rel ↓ 0.424), RMSE (↓ 4.919), and RMSE log (↓ 0.079) on the SCARED dataset, while attaining the highest δ<1.25 (↑ 0.976). As shown in Table 3, CoDepth consistently ranks first across all metrics on the SERV-CT dataset, achieving the lowest Abs Rel (↓ 0.093), Sq Rel (↓ 1.364), RMSE (↓ 9.886), and RMSE log (↓ 0.120), along with the highest δ<1.25 (↑ 0.919). Furthermore, as shown in Table 4, CoDepth ranks first in nearly all metrics on the Hamlyn dataset, including the lowest Abs Rel (↓ 0.066), Sq Rel (↓ 0.003), RMSE (↓ 0.040), and RMSE log (↓ 0.092). As shown in Table 5, CoDepth achieves the lowest Abs Rel (↓ 0.222) and the highest δ<1.25 (↑ 0.659) on the C3VD dataset, ranking first in most evaluated metrics. The results across these four datasets demonstrate CoDepth’s strong performance in diverse endoscopic scenarios (including varying anatomical regions, lighting conditions, and endoscope types), highlighting its excellent generalization capability.

As shown in Table 6, CoDepth achieves 17.56 FPS on an NVIDIA RTX A5000, with an average inference time of 57.0 ms per frame, demonstrating a competitive efficiency profile among the compared methods. While it is slower than the lightweight discriminative baseline AF-SfMLearner (78.85 FPS, 12.7 ms), it remains substantially faster than the diffusion-based MonoDiffusion model (12.49 FPS, 80.0 ms). This result suggests that CoDepth offers a favorable compromise between inference efficiency and model expressiveness.

Another notable strength of CoDepth lies in its parameter-efficient trainable design. Specifically, the reported Trainable Parametersof CoDepth is only 0.970 M, as this metric accounts solely for the trainable parameters in the fusion/adaptation modules. This indicates that the proposed framework introduces only a lightweight learnable component on top of the base estimators, which is desirable for model adaptation and optimization. Although the **Total Params** during inference reaches 33.814 M, this increase is mainly due to the inclusion of two frozen base depth estimation branches together with the fusion modules, rather than to a large trainable parameter budget.

At the same time, CoDepth requires 19.730 G FLOPs, which is higher than AF-SfMLearner and MonoDiffusion. This additional cost arises from the dual-branch inference process and the subsequent fusion operations. Nevertheless, considering its compact trainable core, and intermediate inference speed, CoDepth is more appropriately viewed as an accuracy- and robustness-oriented fusion framework with practical deployment potential, rather than as a purely speed-driven alternative to efficient single-branch baselines.

From a deployment perspective, particularly in robotic visual-servoing scenarios where perception latency directly constrains control bandwidth, closed-loop responsiveness, and real-time constraint handling, faster depth inference is clearly desirable [34,35]. This motivates several promising directions for improving the practical suitability of CoDepth in real-time robotic applications. These include: (1) distilling the current dual-branch architecture into a compact student network; (2) applying structured pruning to eliminate redundant channels or layers in both the base networks and fusion modules. Such improvements may substantially reduce latency while preserving the robustness benefits of the proposed fusion framework.

**Figure 4:**
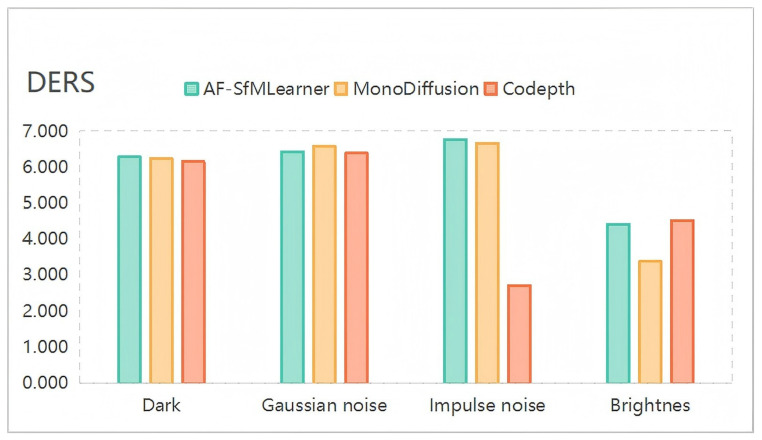
Robustness comparison of CoDepth, MonoDiffusion [8] and AF-SfMLearner [4] with the proposed DERS metric.

To evaluate the robustness of endoscopic depth estimation models, we employed the method described in [36] to create datasets with varying levels of distortion based on the SCARED dataset. The distortions included darkness, Gaussian noise, brightness variations, and impulse noise. The Depth Estimation Robustness Score (DERS) was then calculated to assess model performance under these conditions, with a smaller DERS indicating more robust performance. As illustrated in Figure 4, the results demonstrate that endoscopic depth estimation models exhibit varying sensitivities to different types of corruption. Among the four types of distortion, CoDepth consistently outperforms other methods for most data corruptions, except for brightness corruption.

The inferior performance of CoDepth under brightness corruption likely stems from the fact that the current fusion module mainly exploits inter-branch disagreement as a cue for complementarity. This assumption is less effective when illumination distortion causes both branches to degrade simultaneously, because disagreement no longer reliably reflects branch reliability. Moreover, the confidence module is not explicitly illumination-aware and is built upon frozen base estimators whose intermediate representations remain sensitive to photometric shift. Consequently, the fusion module may reweight corrupted predictions rather than correct them. A promising direction for improvement is to introduce illumination-aware confidence cues, such as brightness statistics, saturation maps, highlight responses, and reprojection residuals, together with illumination-oriented augmentation during training. In addition, lightweight feature adaptation modules and an uncertainty-aware fallback fusion mechanism may further improve robustness under unstable endoscopic lighting.

Beyond reporting aggregate test metrics, we further examine whether the performance gains are consistent across individual test samples. To this end, Table 7 summarizes per-sample statistics on the test set, including the mean and 95% confidence interval of each metric, and reports paired *t*-tests on per-sample metric values between methods.

As shown in Table 7, the proposed CoDepth method demonstrates statistically significant differences compared to MonoDiffusion and AF-SfMLearner across all evaluated metrics (p<0.05, paired *t*-tests).

### 4.3. Qualitative Comparison Results

**Figure 5:**
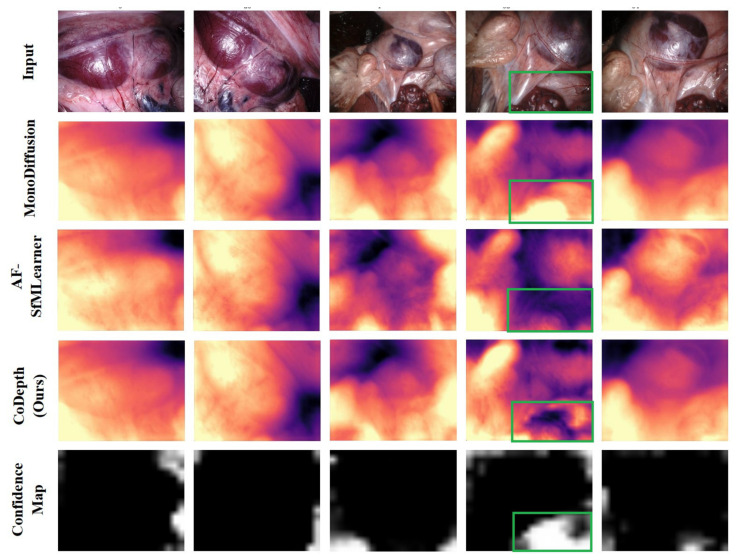
Qualitative depth comparison and confidence map visualization on the SCARED dataset. The discriminative model (AF-SfMLearner [4]) and the generative model (MonoDiffusion [8]) exhibit complementary failure modes in challenging endoscopic scenes. The green framed region corresponds to a complex tissue area with specular reflections and low texture, where MonoDiffusion produces over-smoothed depth while AF-SfMLearner introduces local artifacts. The confidence map (last row) assigns higher confidence to the more reliable prediction in this region, guiding adaptive fusion and resulting in a more stable and consistent depth estimate in CoDepth.

Figure 5 shows that the proposed modules learn meaningful spatial patterns for fusion. The complementary map is mainly activated around tissue boundaries, thin structures, weak-texture regions, and local highlight areas, where the two branches exhibit noticeable disagreement. This indicates that it captures structured discrepancy related to branch-specific failure modes rather than random noise. Confidence maps tend to show the discriminative branch in contour-preserving and geometrically stable regions, whereas the diffusion branch often exhibits higher confidence in homogeneous or locally ambiguous areas. Therefore, the final fusion is not a simple average of two predictions, but a spatially adaptive selection process that explains the superior qualitative and quantitative performance of CoDepth.

### 4.4. Ablation Study

We conducted detailed ablation studies to evaluate the contribution of each component, focusing on the confidence fusion module, multi-model fusion strategy and loss function design.

**Confidence Fusion Module and Multi-Model Fusion Strategy**: To evaluate confidence-guided multi-model fusion, we perform an ablation study with progressively enhanced fusion strategies. ID 1 and ID 2 denote the two single-branch baselines, each obtained by removing one of the two frozen depth estimators and retaining only the discriminative branch or the diffusion branch, respectively, for prediction. ID 3 denotes direct prediction averaging, ID 4 denotes naive feature concatenation with convolutional fusion, ID 5 denotes confidence prediction from concatenated features only, and ID 6 denotes the full CoDepth model.

As shown in Table 8, simply combining the two estimators does not necessarily improve performance. In particular, the naive fusion strategy in ID4 causes a clear degradation and even performs worse than both single-branch baselines. This is because the discriminative and diffusion branches provide heterogeneous representations with different strengths and failure modes: the former is generally more stable in preserving global geometry and sharp boundaries, whereas the latter contributes richer local details but may introduce structurally inconsistent responses in ambiguous regions. Direct concatenation and convolution mix these conflicting cues without modeling branch reliability, which often leads to over-smoothed transitions and error propagation in challenging endoscopic regions such as highlights, weak-texture tissue, and blurred areas. Although ID5 introduces confidence prediction, its effect is still limited because confidence is estimated from already mixed features and thus cannot explicitly characterize the structured disagreement between branches. By contrast, the full model in ID6 first extracts complementary information from prediction discrepancy, then refines it through cross-attention, and finally performs confidence-guided selective fusion. This enables spatially adaptive integration of the two branches and leads to the best performance across all metrics.

**Loss Function Design:** To analyze the contribution of each loss term to the model’s optimization, we designed the following configurations: ID 1 removes the reprojection loss, ID 2 removes the transform consistency loss, ID 3 removes the disparity smooth loss, ID 4 removes the transform smooth loss, ID 5 removes the confidence regularization loss, and ID 6 uses the complete loss function. As shown in Table 9, the best performance is achieved when all loss terms are utilized.

**Weight Parameters of the Loss Function:** To validate the impact of the weight parameters on the results, we designed the following experiment. Since testing all possible combinations of the five parameters would be computationally expensive, we adopted a simplified approach. Initially, each weight is assigned an empirical value and then we swap the values of two weights at a time. Next, we use the One Factor at a Time (OFAT) method to fix the other weights and vary the weight of the loss function to test the impact of changes in the loss function weight. By changing the magnitude of the weights in this way, we observe the effect on the results. The rationale for this design is that the goal of the present ablation is to examine the sensitivity and individual contribution of each loss term in an interpretable manner, rather than to perform exhaustive joint hyperparameter optimization. This protocol enables clearer attribution of performance variations to a specific component and avoids the substantial computational burden associated with searching the full multi-dimensional weight space.

However, OFAT does not account for possible nonlinear interactions among different loss terms. Consequently, the findings in Table 10 should be interpreted as a local component-wise sensitivity analysis, rather than as evidence of a globally optimal weight configuration. A more systematic exploration of coupled hyperparameter effects, for example through factorial design, grid search, or Bayesian optimization, will be pursued in future work.

The weight matrix is shown in Table 10. As shown in Table 11, ID1 represents the results obtained by evaluating with the data from the first row of the weight matrix, ID2 represents the results obtained by evaluating with the data from the second row, ID3 represents the results from the third row, ID4 from the fourth row, and ID5 from the fifth row of the weight matrix. ID 6 to ID 9 represent the results obtained by varying only the weight of the confidence loss function.

**Ablation Study on Confidence Regularization:** To better understand the role of each component in the proposed confidence-guided fusion, we conduct a detailed ablation study focusing on the confidence regularization terms. As discussed in Overall Loss Function, the confidence map is constrained by three complementary objectives: an overlap penalty, an entropy regularization, and a global balance constraint. This study aims to verify whether the confidence map degenerates into a hard binary selector, and to analyze how each regularization term contributes to stable and adaptive fusion.

Specifically, we evaluate several variants by selectively enabling or disabling different confidence constraints: (ID1) using only the overlap penalty, (ID2) using only entropy regularization, (ID3) using only the balance constraint, (ID4) removing entropy regularization, (ID5) removing the balance constraint, (ID6) removing the overlap penalty, and (ID7) the full model with all confidence regularization terms enabled (baseline).

All variants are evaluated on the SCARED dataset under identical training and evaluation settings.

Table 12 shows that relying on a single confidence regularization term is insufficient. Although the overlap-only variant achieves slightly better Abs Rel, it exhibits reduced efficiency and less favorable overall trade-offs. Removing the entropy regularization leads to degraded depth estimation accuracy across all error metrics, indicating that the absence of entropy constraints weakens the effectiveness of confidence modeling. Similarly, removing the balance constraint results in an overall performance drop, suggesting that without explicit regulation, the confidence assignment is more prone to imbalance. In contrast, removing the overlap penalty further affects fusion performance, highlighting the important role of complementary consistency constraints in stabilizing confidence allocation and fusion behavior.

The best overall performance is obtained when all confidence constraints are jointly applied, confirming that the proposed confidence map enables probabilistic and complementary fusion rather than acting as a hard per-pixel selector.

### 4.5. Limitations and Implications for Surgical Robot Automation

Although the proposed CoDepth framework shows strong performance for endoscopic monocular depth estimation, this study cannot serve as the complete technical support for the R&D of automated surgical robots. The current work focuses on the perception layer and is validated mainly on offline dataset benchmarks rather than in closed-loop robotic systems. Therefore, the results should be interpreted as evidence of improved depth perception capability, not as a direct demonstration of surgical autonomy.

Nevertheless, CoDepth can function as a depth perception submodule within automated or semi-automated surgical systems, providing upstream perceptual information for intraoperative navigation, field-of-view optimization, instrument–tissue distance estimation, and risk early warning. Inspired by previous relevant studies [37,38], further practical deployment requires system-level integration and validation with visual servo control, instrument tracking, remote center of motion (RCM) safety constraints, and anti-disturbance control. Future work should additionally evaluate temporal stability, real-time performance, and task-level utility in robotic scenarios such as autonomous camera guidance and safe tool motion.

## 5. Conclusions

The proposed CoDepth framework was designed to exploit the complementary strengths of discriminative and diffusion-based depth estimators. By adaptively weighting complementary predictions according to spatial confidence estimates, the framework improves depth accuracy while mitigating the limitations associated with pseudo-ground-truth supervision. Across four public endoscopic datasets (SCARED, Hamlyn, C3VD, and SERV-CT), CoDepth consistently improved several key depth estimation metrics and demonstrated robust cross-domain generalization.

Several challenges remain before practical deployment, including computational efficiency, temporal consistency, and integration with robotic control systems. In particular, although CoDepth provides useful upstream depth cues for surgical scene understanding, navigation assistance, and safety-related perception, it does not yet constitute a complete technical solution for automated surgical robots. Further progress will require joint validation with instrument tracking, visual servoing, motion safety constraints, and closed-loop control under realistic intraoperative conditions.

## Figures and Tables

**Table 1:** Relationship between the internal components in CoDepth and the experimental baselines.

Internal Name	Role in CoDepth	Relation to Baseline	Trainability	Outputs Used in Fusion
Discriminative model	Base estimator 1	AF-SfMLearner-based discriminative estimator adopted as the frozen structural predictor	Frozen	Disparity $d^{\mathrm{disc}}$, multi-scale features $F_{\mathrm{disc}}^{\left(k\right)}$
Diffusion model	Base estimator 2	MonoDiffusion-based generative estimator adopted as the frozen detail-aware predictor	Frozen	Disparity $d^{\mathrm{diff}}$, multi-scale features $F_{\mathrm{diff}}^{\left(k\right)}$
Fusion module	Proposed module	Original component of this work	Trainable	Complementary map Δ, complementary feature $F_{c}$, attention-enhanced feature $F_{a}$, confidence map *C*, fused depth ${\hat{D}}_{t}$

**Table 2:** Quantitative depth comparison on the SCARED dataset.

Method	Abs Rel	Sq Rel	RMSE	RMSE Log	δ<1.25
SFMLearner [13]	0.079	0.879	6.896	0.110	0.947
Fang et al. [31]	0.078	0.794	6.794	0.109	0.946
DeFeat-Net [32]	0.077	0.792	6.688	0.108	0.941
SC-SfMLearner [15]	0.068	0.645	5.988	0.097	0.957
Monodepth2 [14]	0.071	0.590	5.606	0.094	0.953
Endo-SfM [33]	0.062	0.606	5.726	0.093	0.957
AF-SfMLearner [4]	0.059	0.435	4.925	0.082	0.974
MonoDiffusion [8]	0.057	0.474	5.218	0.083	0.973
Yang et al. [20]	0.062	0.558	5.585	0.090	0.962
**CoDepth (Ours)**	**0.055**	**0.424**	**4.919**	**0.079**	**0.976**

The best results are in bold. The second-best results are underlined. Values represent primary results from a single test run; numbers are rounded for readability.

**Table 3:** Quantitative depth comparison on the SERV-CT dataset.

Method	Abs Rel	Sq Rel	RMSE	RMSE Log	δ<1.25
SfMLearner [13]	0.151	3.917	17.451	0.191	0.779
Fang et al. [31]	0.149	3.099	15.564	0.188	0.787
DeFeat-Net [32]	0.114	1.946	12.588	0.153	0.873
SC-SfMLearner [15]	0.117	2.015	12.415	0.148	0.852
Monodepth2 [14]	0.123	2.205	12.927	0.152	0.856
Endo-SfM [33]	0.116	2.014	12.493	0.143	0.864
AF-SfMLearner [4]	0.102	1.632	11.092	0.131	0.898
MonoDiffusion [8]	0.097	1.560	10.581	0.126	0.904
**CoDepth (Ours)**	**0.093**	**1.364**	**9.886**	**0.120**	**0.919**

The best results are in bold. The second-best results are underlined. Values represent primary results from a single test run; numbers are rounded for readability.

**Table 4:** Quantitative depth comparison on the Hamlyn dataset.

Method	Abs Rel	Sq Rel	RMSE	RMSE Log	δ<1.25
AF-SfMLearner [4]	0.079	0.004	0.042	0.106	**0.977**
MonoDiffusion [8]	0.068	0.003	0.042	0.094	0.974
**CoDepth (Ours)**	**0.066**	**0.003**	**0.040**	**0.092**	0.975

The best results are in bold. The second-best results are underlined. Values represent primary results from a single test run; numbers are rounded for readability.

**Table 5:** Quantitative depth comparison on the C3VD dataset.

Method	Abs Rel	Sq Rel	RMSE	RMSE Log	δ<1.25
AF-SfMLearner [4]	0.225	1.744	6.009	0.256	0.624
MonoDiffusion [8]	0.224	**1.664**	**5.670**	**0.254**	0.656
**CoDepth (Ours)**	**0.222**	1.683	5.676	**0.254**	**0.659**

The best results are in bold. The second-best results are underlined. Values represent primary results from a single test run; numbers are rounded for readability.

**Table 6:** Performance comparison on the SCARED dataset.

Method	FPS	Inference Time (ms)	FLOPs	Trainable Parameters	Total Params
AF-SfMLearner [4]	78.85	12.7	5.359 G	14.842 M	14.842 M
MonoDiffusion [8]	12.49	80.0	8.722 G	3.162 M	18.003 M
CoDepth (Ours)	17.56	57.0	19.730 G	0.970 M	33.814 M

**Table 7:** Quantitative comparison on the SCARED dataset with statistics computed from per-sample results. Values in this table are computed as per-sample means on the test set. Slight discrepancies from Table 2 reflect that Table 2 reports single-run results rounded for presentation.

Method	Abs Rel	95% CI	Sq Rel	95% CI	RMSE	95% CI	RMSE log	95% CI	δ<1.25	95% CI
MonoDiffusion [8]	0.0575	[0.0556, 0.0594]	0.4838	[0.4450, 0.5226]	5.1924	[4.9273, 5.4575]	0.0838	[0.0807, 0.0869]	0.9715	[0.9685, 0.9744]
AF-SfMLearner [4]	0.0591	[0.0572, 0.0610]	0.4352	[0.4064, 0.4639]	4.9255	[4.7289, 5.1221]	0.0816	[0.0790, 0.0843]	0.9738	[0.9707, 0.9768]
CoDepth (Ours)	0.0553	[0.0536, 0.0571]	0.4249	[0.3930, 0.4568]	4.9193	[4.6946, 5.1439]	0.0793	[0.0766, 0.0820]	0.9756	[0.9728, 0.9785]

CI is the abbreviation of confidence intervals. The paired *p*-values of our method and the compared methods are less than 0.05 on all metrics. Values are reported as the mean and 95% confidence intervals (CI) computed from per-sample results on the test set. Paired *t*-tests performed on per-sample metric values under identical test samples yield *p*-values below 0.05 for all reported metrics, indicating that the performance differences between CoDepth and the compared methods are statistically significant. CoDepth achieves the best mean performance on Abs Rel, Sq Rel, RMSE log, and δ<1.25, while remaining competitive on RMSE. The relatively narrow confidence intervals indicate stable performance across test samples.

**Table 8:** Ablation study on SCARED datasets for Confidence Fusion Module.

ID	Abs Rel	Sq Rel	RMSE	RMSE Log	δ<1.25
1	0.057	0.445	5.025	0.081	0.974
2	0.057	0.446	5.027	0.081	0.974
3	0.057	0.442	5.008	0.081	0.974
4	0.107	1.398	8.764	0.147	0.892
5	0.056	0.438	4.984	0.081	0.975
6	**0.055**	**0.424**	**4.919**	**0.079**	**0.976**

Bold indicates the best results.

**Table 9:** Ablation study on SCARED datasets for Loss Function Design.

ID	Abs Rel	Sq Rel	RMSE	RMSE Log	δ<1.25
1	0.057	0.455	5.071	0.082	0.974
2	0.057	0.443	5.011	0.081	0.974
3	0.057	0.443	5.009	0.081	0.974
4	0.057	0.450	5.054	0.081	0.974
5	0.057	0.451	5.056	0.082	0.974
6	**0.055**	**0.424**	**4.919**	**0.079**	**0.976**

Bold indicates the best results.

**Table 10:** Weight matrix of loss function.

ID	$L_{D-S}$	$L_{R-L}$	$L_{T-C}$	$L_{T-S}$	$L_{\mathrm{conf}}$
1	0.100	0.500	0.200	0.020	0.010
2	0.500	0.100	0.200	0.020	0.010
3	0.100	0.200	0.500	0.020	0.010
4	0.100	0.020	0.200	0.500	0.010
5	0.100	0.010	0.200	0.020	0.500
6	0.500	0.100	0.200	0.020	0.500
7	0.500	0.100	0.200	0.020	0.100
8	0.500	0.100	0.200	0.020	0.200
9	0.500	0.100	0.200	0.020	1.000
10	0.500	1.000	0.200	0.020	0.010

**Table 11:** Ablation study on SCARED datasets for weight matrix of Loss Function Design.

ID	Abs Rel	Sq Rel	RMSE	RMSE Log	δ<1.25
1	0.056	0.464	5.133	0.082	0.974
2	0.056	0.460	5.120	0.082	0.974
3	0.056	0.441	5.011	0.080	0.975
4	0.056	0.456	5.088	0.081	0.975
5	0.056	0.464	5.130	0.082	0.974
6	0.056	0.464	5.131	0.082	0.974
7	0.056	0.465	5.133	0.082	0.974
8	0.056	0.464	5.131	0.082	0.974
9	0.056	0.464	5.133	0.082	0.974
10	**0.055**	**0.424**	**4.919**	**0.079**	**0.976**

Bold indicates the best results.

**Table 12:** Ablation study on confidence regularization components on the SCARED dataset.

Method	Abs Rel	Sq Rel	RMSE	δ<1.25
conf_overlap_only	**0.055**	0.437	4.982	0.975
conf_entropy_only	0.057	0.448	5.041	0.974
conf_balance_only	0.057	0.451	5.058	0.974
w/o entropy	0.057	0.450	5.054	0.974
w/o balance	0.057	0.443	5.011	0.974
w/o overlap	0.057	0.445	5.023	0.974
**Baseline (Full)**	**0.055**	**0.424**	**4.919**	**0.976**

Bold indicates the best results.

## Data Availability

The data that support the findings of this study were derived from the following resources available in the public domain: SCARED dataset (https://endovissub2019-scared.grand-challenge.org) (accessed on 8 October 2025); SERV-CT dataset (https://www.ucl.ac.uk/interventional-surgical-sciences/weiss-open-research/weiss-open-data-server/serv-ct) (accessed on 8 October 2025); Hamlyn dataset (https://davidrecasens.github.io/EndoDepthAndMotion/) (accessed on 8 October 2025); C3VD dataset (https://durrlab.github.io/C3VD/) (accessed on 8 October 2025).
